# Evaluation of Xpert MTB/RIF and GenoType MTBDRplus for the Detection of Rifampicin-Resistant Tuberculosis: A Cross-Sectional Diagnostic Accuracy Study at a Tertiary Care Center in India

**DOI:** 10.7759/cureus.91825

**Published:** 2025-09-08

**Authors:** Anushka Soni, Richa Mishra, Alok Nath, Prerna Kapoor

**Affiliations:** 1 Microbiology, Sanjay Gandhi Postgraduate Institute of Medical Sciences, Lucknow, IND; 2 Pulmonary Medicine, Sanjay Gandhi Postgraduate Institute of Medical Sciences, Lucknow, IND; 3 Internal Medicine, Sanjay Gandhi Postgraduate Institute of Medical Sciences, Lucknow, IND

**Keywords:** cbnaat/ xpert/ rif assay, line probe assay (lpa), mycobacterium tuberculous, pulmonary, rr-tb (rifampicin-resistant tuberculosis)

## Abstract

Background

Accurate and timely detection of *Mycobacterium tuberculosis* (MTB) and associated drug resistance is vital for effective tuberculosis (TB) control, especially in high-burden countries like India. In recent years, molecular assays have significantly improved TB diagnostics. This study evaluates and compares the diagnostic accuracy of the Xpert MTB/RIF assay and the GenoType MTBDRplus version 2.0 line probe assay (LPA) in identifying MTB and rifampicin resistance in both pulmonary and extrapulmonary clinical specimens.

Methodology

Over an 18-month period, 500 clinical specimens, comprising 257 pulmonary and 243 extrapulmonary samples, were analyzed using Ziehl-Neelsen staining, Lowenstein-Jensen culture, and the Xpert MTB/RIF assay. All samples positive for MTB by either culture or Xpert (n = 124) were further assessed using LPA. Cases with discordant rifampicin resistance findings between molecular assays were validated using phenotypic drug susceptibility testing (DST) using the MGIT 960 SIRE system.

Results

Among the 124 MTB-positive cases, pulmonary samples accounted for 90 (72.6%), and extrapulmonary samples accounted for 34 (27.4%). Xpert MTB/RIF detected MTB in 121 cases, showing a sensitivity of 95.3% and specificity of 86.9% compared to culture. Culture positivity was 48 (53.3%) in pulmonary and 16 (44.4%) in extrapulmonary specimens. Valid LPA results were obtained in 119 samples, with MTB detected in 86 cases, yielding an overall sensitivity of 72.3%. Detection rates were higher in smear-positive (48, 92.3%) compared to smear-negative samples (38, 52.7%). Rifampicin resistance was identified by Xpert in 28 (22.5%) cases, with 7 (8.1%) instances of discordance between Xpert and LPA results. Of these, five were concordant with LPA and two with Xpert upon phenotypic DST.

Conclusions

The Xpert MTB/RIF assay demonstrated excellent sensitivity, particularly in smear-negative and extrapulmonary samples. LPA showed better concordance with phenotypic DST for rifampicin resistance but was less effective in smear-negative cases. These findings highlight the complementary roles of molecular and phenotypic methods in enhancing the diagnosis of TB and detecting resistance.

## Introduction

Tuberculosis (TB), caused by *Mycobacterium tuberculosis* (MTB), remains a critical public health issue, particularly in high-burden countries like India. According to the World Health Organization (WHO) Global TB Report 2023, India accounts for the largest number of TB cases globally, including a considerable burden of multidrug-resistant TB (MDR-TB) [1]. Early detection and accurate identification of drug resistance are crucial for effective disease management and preventing transmission. Traditionally, TB diagnosis in resource-limited settings has relied on smear microscopy using Ziehl-Neelsen (ZN) staining and culture on Lowenstein-Jensen (LJ) medium. While these methods are foundational, they are hampered by limitations. Smear microscopy lacks sensitivity, particularly in extrapulmonary and paucibacillary cases, and culture, although more sensitive, is time-consuming and requires advanced laboratory infrastructure [2,3]. In recent years, molecular assays have significantly improved TB diagnostics. The GeneXpert MTB/RIF assay, a cartridge-based nucleic acid amplification test endorsed by the WHO, enables the rapid detection of MTB DNA, along with rifampicin resistance, by targeting mutations within the rifampicin resistance-determining region (RRDR) of the *rpoB* gene. With results available in under two hours and minimal technical requirements, this assay has been widely adopted in national TB programs, especially in decentralized settings [4,5]. Real‑world evaluations have demonstrated pooled sensitivities of about 98% in smear‑positive cases (specificity ~99%) and around 67-78% in smear‑negative, culture‑positive specimens (specificity ~99%) [5-7]. Another widely used molecular tool is the GenoType MTBDRplus version 2.0 assay, a line probe assay (LPA) that detects resistance not only to rifampicin but also to isoniazid by identifying mutations in the *rpoB*, *katG*, and *inhA* genes [8,9]. While the diagnostic accuracy of LPA is well established in smear-positive pulmonary TB cases, its utility in smear-negative and extrapulmonary specimens remains underexplored in the Indian context [10]. Despite the availability of these tools, there is limited evidence from India assessing and comparing the performance of GeneXpert and LPA v2.0 across diverse clinical sample types. especially smear‑negative and extrapulmonary cases [3,9-11]. This gap is particularly important considering that microbiological testing is often unavailable in peripheral health centers and smaller hospitals, where empirical treatment is common due to diagnostic limitations [12].

This cross-sectional diagnostic accuracy study was designed with the following objectives: Primary objective: to evaluate and compare the diagnostic accuracy of Xpert MTB/RIF and GenoType MTBDRplus v2.0 for detecting MTB and rifampicin resistance in pulmonary and extrapulmonary samples. Secondary objectives: to (a) assess their diagnostic yield relative to smear microscopy and culture, (b) investigate concordance between molecular results and phenotypic drug susceptibility testing (DST), and (c) explore their potential clinical utility in improving TB diagnostic algorithms in high-burden, resource-constrained settings. This study aims to provide critical insights for optimizing TB diagnostic algorithms in high-burden, resource-limited settings.

## Materials and methods

Study design and setting

This cross-sectional, prospective diagnostic accuracy study was conducted over an 18-month period from April 2017 to October 2018 in the Mycobacteriology Section, Department of Microbiology, Sanjay Gandhi Postgraduate Institute of Medical Sciences (SGPGIMS), Lucknow, India. The study population included 500 clinically suspected TB cases, comprising both pulmonary and extrapulmonary presentations, including suspected MDR-TB.

Ethical considerations

Before initiation, ethical clearance was obtained from the Institutional Ethics Committee of SGPGIMS, Lucknow (approval number: 2017-37-IMP-EXP). Written informed consent was obtained from all patients or their guardians before specimen collection.

Inclusion and exclusion criteria

Consecutive specimens were collected from patients (both inpatient and outpatient) with clinical and/or radiological suspicion of TB, including symptoms such as persistent cough >2 weeks, fever, weight loss, or imaging findings suggestive of TB. Samples from patients already on anti-TB treatment for >2 weeks, specimens with insufficient volume, and patients with known HIV co-infection were excluded.

Specimen handling and processing

All specimens were processed in a Biosafety Level 2 cabinet using appropriate personal protective equipment. Each sample was split into two aliquots, i.e., one for GeneXpert MTB/RIF testing and the other for culture and LPA. Pulmonary samples were decontaminated using the NALC-NaOH method; extrapulmonary specimens, including fine-needle aspiration cytology (FNAC) and body fluids, were processed directly. Smears were stained using ZN staining for acid-fast bacilli (AFB) detection. Specimens were inoculated onto LJ medium and incubated at 37°C for up to eight weeks. Cultures were monitored weekly, and positive isolates were confirmed as MTB complex (MTBC) using immunochromatographic methods.

Molecular and culture-based diagnostic methods

GeneXpert MTB/RIF Assay

The GeneXpert MTB/RIF assay (Cepheid, Sunnyvale, CA, USA; G4 cartridge version) was performed following manufacturer instructions. Processed specimens were incubated with the sample reagent and loaded into the cartridge. Results were generated within two hours, indicating the presence of MTB and rifampicin resistance [4,5].

GenoType MTBDRplus Version 2.0 (LPA)

The LPA (Hain Lifescience GmbH, Germany) detects MTB and resistance to rifampicin and isoniazid by identifying mutations in the *rpoB*, *katG*, and *inhA* genes. The procedure included DNA extraction, multiplex polymerase chain reaction, and reverse hybridization on nitrocellulose strips. Results were visually interpreted as per manufacturer guidelines [8,9].

Phenotypic DST

Detailed procedural steps for phenotypic DST using the MGIT 960 SIRE system are provided in the Appendices [13].

Controls

MTB H37Rv was used as a positive control in all DST runs.

## Results

During the 18-month study period, a total of 500 clinical samples were included, comprising 257 pulmonary and 243 extrapulmonary specimens. Each sample underwent simultaneous processing for ZN staining for AFB, inoculation on LJ medium, and analysis using the Xpert MTB/RIF assay.

**Figure 1:**
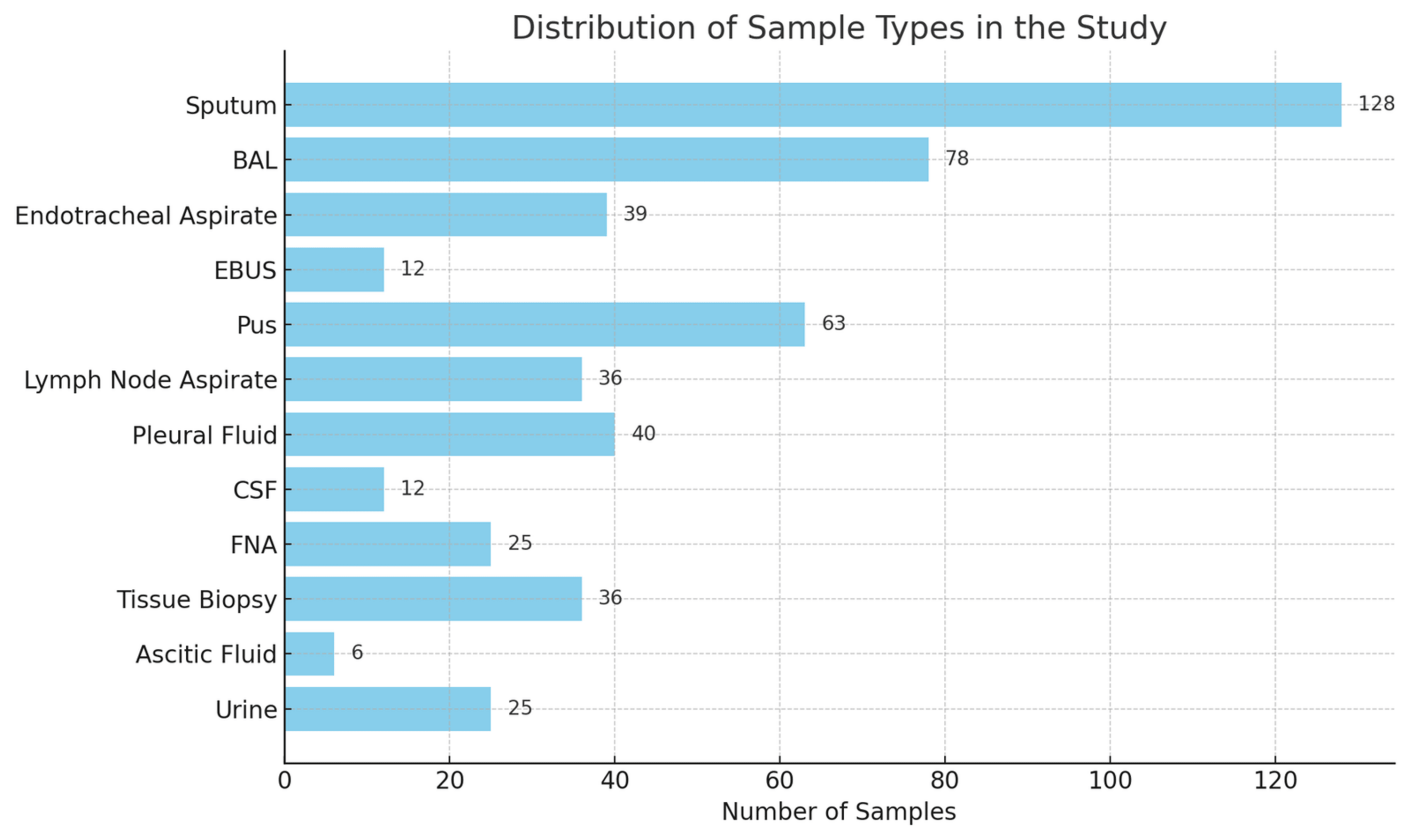
Distribution of samples included in the study (n = 500). BAL = bronchoalveolar lavage; EBUS = endoscopic bronchial ultrasound-guided; CSF = cerebrospinal fluid; FNA = fine-needle aspiration

The study encompassed a wide range of sample types, namely, sputum (n = 128), bronchoalveolar lavage (BAL) (n = 78), endotracheal aspirate (n = 39), endoscopic bronchial ultrasound-guided (EBUS) samples (n = 12), pus (n = 63), lymph node aspirate (n = 36), pleural fluid (n = 40), cerebrospinal fluid (CSF) (n = 12), fine-needle aspirate (FNA) (n = 25), tissue biopsy (n = 36), ascitic fluid (n = 6), and urine (n = 25) (Figure 1).

**Figure 2:**
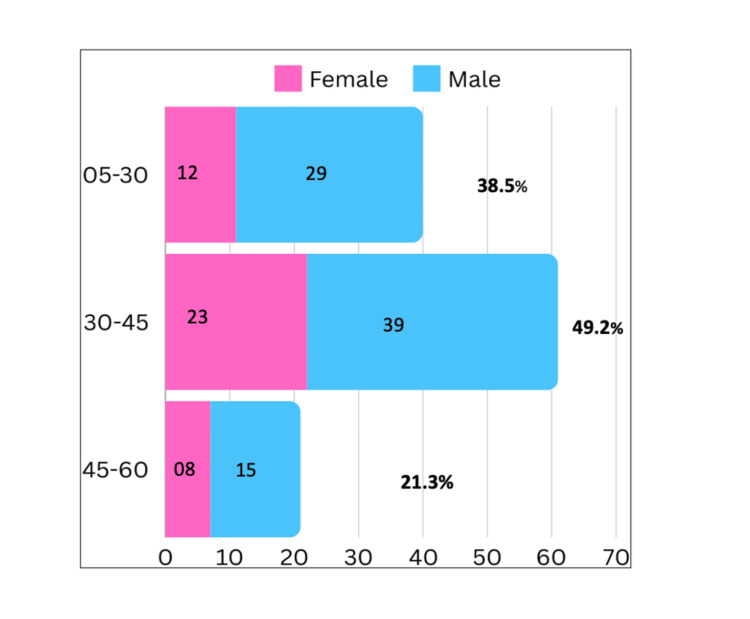
Age Group Stratification of Positive Cases

Among the 126 individuals identified as MTB positive by any of the employed diagnostic assays, 83 (65.8%) were male and 43 (34.1.0%) were female. The median age of the affected individuals was 32 years. A significant proportion (62, 49.2%) of cases belonged to the 30-45-year age group (Figure 2).

Of the 126 MTB-positive cases, 90 (71.4%) were pulmonary in origin, while 36 (28.6%) were extrapulmonary. Smear and culture positivity rates among pulmonary samples were 41 (45.5%) and 48 (53.3%), respectively, whereas extrapulmonary samples showed positivity rates of 11 (30.5%) for smear microscopy and 16 (44.4%) for culture (Table 1).

**Table 1:** Distribution of smear and culture positivity in pulmonary and extrapulmonary samples.

Type of sample	Smear positive, n (%)	Culture positive, n (%)
Pulmonary (n = 90)	41 (45.5%)	48 (53.3%)
Extrapulmonary (n = 36)	11 (30.5.%)	16 (44.4%)
Total (n = 126)	52 (41.2%)	64 (50.7%)

All 64 culture-positive samples were further tested using the MPT64 antigen detection assay, which yielded positive results in 59 samples, while two were negative, and three cultures were contaminated.

The GeneXpert MTB/RIF assay detected MTB in 121 out of 124 total positive cases. Notably, the assay failed to detect MTB in three samples that were culture-positive, suggesting a small proportion of false negatives. The overall MTB positivity rate in the study population was 124/500 (24.8%). Using culture as the reference standard, the sensitivity of the GeneXpert assay was calculated to be 95.3% (61/64), and the specificity was 86.9% (397/457). These findings highlight the high diagnostic accuracy of GeneXpert in detecting MTB, including its utility in both pulmonary and extrapulmonary samples.

All 124 out of 500 samples that tested positive for MTB by either GeneXpert or culture were subjected to the GenoType MTBDRplus version 2.0 LPA, performed directly on a second aliquot of each specimen. Among smear-positive samples (n = 52), MTB was detected by LPA in 48 cases, yielding a detection rate of 48/52 (92.30%). Among smear-negative samples (n = 72), MTB was detected in 38 cases, corresponding to a positivity rate of 38/72 (52.57%). LPA failed to detect MTB in four smear-positive and 29 smear-negative samples. Additionally, five smear-negative samples yielded invalid LPA results and were excluded from the final analysis.

After excluding the invalid results, LPA results were available for 119 MTB-positive samples. Of these, LPA correctly identified MTB in 86 cases (true positives) and failed to detect it in 33 cases (false negatives). This yielded an overall sensitivity of 72.27% for LPA in our cohort. Specificity could not be assessed in this study, as LPA was not performed on MTB-negative samples. The diagnostic performance of smear microscopy, GeneXpert MTB/RIF assay, LPA, and culture was evaluated across both groups.

Pulmonary samples (n = 90)

The pulmonary specimens analyzed in this study comprised sputum (n = 34), BAL (n = 44), endotracheal aspirates (n = 7), and EBUS aspirates (n = 5). Smear microscopy yielded positive results in 41 of the 90 pulmonary samples (45.5%), with the highest positivity observed in sputum (17/34, 50%) and BAL samples (19/44, 43.2%). The GeneXpert MTB/RIF assay demonstrated a high detection rate, identifying MTB in 89 out of 90 pulmonary samples (98.9%). The sole GeneXpert-negative result was observed in a BAL specimen. The LPA was positive in 64/90 (71.1%) pulmonary samples, with the highest detection rates seen in sputum (25/34, 73.5%) and BAL (33/44, 75%) specimens. Mycobacterial culture was positive in 48/90 (53.3%) pulmonary samples, with the majority of culture-confirmed cases obtained from sputum (22/34, 64.7%) and BAL (20/44, 45.4%). The remaining 42/90 (46.6%) pulmonary samples were culture-negative (Table 2).

**Table 2:** Distribution of the results of microscopy, GeneXpert MTB/RIF assay, LPA, and culture for pulmonary samples (n = 90/124). BAL = bronchoalveolar lavage; EBUS = endoscopic bronchial ultrasound-guided; LPA = line probe assay

Pulmonary samples	Smear positive	Smear negative	Xpert positive	LPA positive	Culture positive	Culture negative
Sputum (n = 34)	17	17	34	25	22	12
BAL (n = 44)	19	25	43	33	20	24
Endotracheal aspirate (n = 7)	3	4	7	4	4	3
EBUS (n = 5)	2	3	5	2	2	3
Total (n = 90)	41	49	89	64	48	42

Extrapulmonary samples (n = 34)

The extrapulmonary specimens included pus (n = 11), lymph node aspirates (n = 10), CSF (n = 3), body fluids (n = 4), tissue biopsies (n = 3), and FNAC samples (n = 3). Smear microscopy was positive in 11 out of 34 samples (32.3%), reflecting the inherent limitations of smear-based detection in extrapulmonary TB due to its typically low bacillary load. The GeneXpert MTB/RIF assay detected MTB in 32 of the 34 extrapulmonary specimens (94.1%). Two samples, i.e., one biopsy and one body fluid, tested negative by GeneXpert, which were smear negative. The LPA demonstrated positivity in 22/34 (64.7%), with the highest detection observed in FNAC (3/3, 100%), CSF (3/3, 100%), and pus samples (7/11, 63.6%). Mycobacterial culture yielded positive results in 16/34 extrapulmonary samples (47.0%), while the remaining 18/34 (52.9%) samples were culture negative (Table 3).

**Table 3:** Distribution and results of microscopy, Xpert MTB/RIF assay, LPA, and culture for extrapulmonary samples (n = 34/124). CSF = cerebrospinal fluid; FNAC = fine-needle aspiration cytology; LPA = line probe assay

Extrapulmonary samples	Smear positive	Smear negative	Xpert positive	LPA positive	Culture positive	Culture negative
Pus (n =11)	4	7	11	7	4	7
Lymph node aspirate (n = 10)	3	7	10	6	4	6
CSF (n = 3)	1	2	3	3	2	1
Body fluid (n = 4)	1	3	3	2	2	2
Biopsy (n = 3)	1	2	2	1	2	2
FNAC (n = 3)	1	2	3	3	2	1
Total (n = 34)	11	23	32	22	16	18

Rifampicin resistance detection and concordance between molecular assays

Out of 124 clinical specimens confirmed positive for MTB by GeneXpert or culture, the Xpert MTB/RIF assay detected rifampicin resistance in 28 cases, representing an overall resistance rate of 28/124 (22.5%). When stratified by specimen type, rifampicin resistance was observed in 22/90 (24.4%) pulmonary samples and 6/34 (17.6%) extrapulmonary samples.

The GenoType MTBDRplus version 2.0 LPA was interpretable for rifampicin resistance in 86 of the MTB-positive cases after excluding uninterpretable or invalid results. Among these, 7/86 (8.1%) cases demonstrated discordance in rifampicin resistance results between Xpert MTB/RIF and LPA. These included specimens from sputum (n = 2), BAL (n = 1), pus (n = 1), lymph node aspirate (n = 2), and tissue biopsy (n = 1).

All discordant cases were further evaluated using phenotypic DST on the MGIT 960 SIRE system, with MTB H37Rv as the control strain. Final classification of rifampicin resistance was made based on concordance with phenotypic DST results. Among the seven discordant samples, five were concordant with the LPA results, while two were consistent with GeneXpert, indicating potential false negatives by LPA. These discrepant cases included both pulmonary (sputum, BAL) and extrapulmonary (pus, lymph node aspirate, tissue biopsy) specimens, reflecting the complexities involved in diagnosing rifampicin resistance in samples with low bacillary load or heteroresistance.

The detection of inconsistencies between molecular methods highlights the importance of follow-up testing with phenotypic assays, especially in rifampicin resistance diagnosis, where treatment decisions critically depend on accurate susceptibility results.

## Discussion

This study evaluated the diagnostic performance of the GeneXpert MTB/RIF and GenoType MTBDRplus version 2.0 (LPA) assays for detecting MTB and rifampicin resistance in both pulmonary and extrapulmonary specimens. The GeneXpert assay achieved excellent sensitivity for pulmonary samples and demonstrated high diagnostic yield overall, reaffirming its standing as a robust frontline tool for rapid MTB and resistance detection [3,14]. Meanwhile, LPA provided rapid detection of rifampicin resistance, particularly in smear-positive specimens, though its sensitivity dropped significantly in smear-negative cases, indicating limitations in paucibacillary contexts [10,15].

Diagnostic gaps in rural and peripheral settings

In India’s rural regions and smaller healthcare centers, microbiological diagnostics such as AFB smear microscopy and culture-based DST are frequently unavailable, compelling clinicians to rely on empirical treatment based on clinical and radiological findings. In our study, smear positivity was observed in 52/124 (41.2%) MTB-positive cases, with 41 (45.5%) from pulmonary and 11 (30.5%) from extrapulmonary specimens. This rate is lower than the 70.3% positivity reported by Rufai et al. but aligns with several other Indian studies documenting lower detection in cases with a high proportion of smear-negative or extrapulmonary TB [10,16,17].

The limited sensitivity of smear microscopy, particularly in paucibacillary forms of TB, is well recognized. The technique requires bacterial loads of 5,000-10,000 CFU/mL for detection, making it poorly suited for extrapulmonary or smear-negative pulmonary cases [9]. Another cohort involving 200 smear-negative pulmonary and extrapulmonary cases showed a culture positivity rate of just 7% [18]. This stark discrepancy underscores the diagnostic blind spots inherent in smear-only approaches and emphasizes the essential role of culture or molecular assays for comprehensive TB detection.

While culture remains the gold standard for diagnosing active pulmonary TB, capable of detecting as few as 10-100 CFU/mL and yielding isolates for DST [19]. Our observed culture positivity rates of 48/90 (53.3%) in pulmonary and 16/34 (47%) in extrapulmonary specimens exceeded figures reported in earlier Indian studies [20,21]. Notably, a recent report by Kanade et al. analyzing bronchoscopic specimens found culture positivity rates around 22%, substantially lower than ours, suggesting that our elevated detection likely reflects inclusion of patients with strong clinical or radiological TB evidence, thereby enriching the cohort for culture-confirmed cases [21].

Diagnostic performance of GeneXpert MTB/RIF

The GeneXpert MTB/RIF assay demonstrated a high detection rate across diverse clinical specimens, identifying 121 out of 126 MTB-positive cases (95.3% sensitivity and 86.9% specificity against culture). Its superior diagnostic yield in pulmonary samples (89/90, 98.9%) and substantial positivity in extrapulmonary cases (32/34, 94.1%) underscore its utility as a frontline diagnostic tool, consistent with previous reports emphasizing its robustness even in smear-negative and paucibacillary samples [5,20]. The assay’s rapid turnaround time and ease of use make it particularly valuable in high-burden, resource-limited settings.

Diagnostic performance of LPA in the study cohort

This GenoType MTBDRplus version 2.0 LPA on 124 MTB-positive clinical specimens identified via GeneXpert or culture. After excluding five invalid results from smear-negative cases, LPA was successfully performed on 119 samples, detecting MTB in 86, resulting in an overall sensitivity of 72.3%. Sensitivity was markedly higher in smear-positive specimens (92.3%), but declined to 52.6% in smear-negative samples. These findings align with previous reports demonstrating LPA’s strong performance in high-burden cases but reduced sensitivity in paucibacillary specimens [10,22]. A recent study further corroborates these results, reporting a sensitivity of 47.4% for LPA in smear-negative pulmonary TB [17]. This highlights the assay’s limited reliability in low bacillary load conditions. While LPA remains a valuable tool for rapid detection in smear-positive TB, it should be used alongside molecular amplification tests or culture to improve diagnostic accuracy in smear-negative and extrapulmonary cases.

Rifampicin resistance detection and assay concordance

In our study, rifampicin resistance was identified by the Xpert MTB/RIF assay in 28 out of 124 (22.5%) MTB-positive specimens. Resistance was more common among pulmonary samples (22/90, 24.4%), compared to extrapulmonary specimens (6/34, 17.6%). These findings align with prior reports from high-burden countries, though they are higher than national prevalence estimates, which often range between 10% and 15% for rifampicin resistance in new and previously treated TB cases [1,23]. The elevated rates in our cohort may reflect a tertiary care referral bias, where patients with suspected or known drug resistance are more frequently evaluated.

Rifampicin resistance was further analyzed in 86 cases using the GenoType MTBDRplus v2.0 assay after excluding invalid results. Among these, 7/86 cases (8.1%) showed discordance between Xpert and LPA results. These included a variety of specimen types, namely, sputum, BAL, pus, lymph node aspirate, and tissue biopsy. All discordant cases were resolved through phenotypic DST using the MGIT 960 SIRE system, with MTB H37Rv as a reference strain.

Discordance between Xpert and LPA is a recognized phenomenon, typically attributed to mutations outside the RRDR, heteroresistance, and low bacterial load, especially in extrapulmonary or smear-negative samples [24,25]. Published studies have reported Xpert-LPA discordance rates of 5%-15%, consistent with our findings [16]. For instance, Van Deun et al. highlighted that LPA tends to detect more resistance mutations when compared with Xpert, particularly in samples harboring “disputed” *rpoB* mutations not always captured by Xpert probes [26].

These observations reinforce the clinical importance of phenotypic DST as a confirmatory method, particularly when molecular assays produce discrepant results. Treatment decisions based solely on one molecular platform may risk under- or overtreatment in rifampicin-resistant TB, especially when resistance is borderline or mutations are rare.

Limitations

This study has several limitations. It was conducted at a single tertiary care center, which may not reflect the broader epidemiological trends in the general population. This study included a limited number of pediatric patients and excluded individuals with HIV co-infection, although these groups often present unique diagnostic challenges due to lower bacillary loads. Furthermore, sequencing of discordant isolates was not performed, which could have provided insights into rare resistance mutations or mixed infections. Lastly, LPA was not performed on MTB-negative samples, precluding specificity assessment for this assay.

## Conclusions

This study confirms that Xpert MTB/RIF is a rapid and highly sensitive assay for diagnosing TB, demonstrating strong performance even in smear-negative and extrapulmonary samples. In comparison, the LPA showed greater agreement with phenotypic DST, reinforcing its reliability for confirming rifampicin resistance. Together, these results emphasize the complementary roles of molecular diagnostics, although findings should be interpreted cautiously due to the single-center design and the referral bias inherent to a tertiary care facility, which may partly explain the elevated rifampicin resistance rate observed. A stepwise diagnostic strategy, incorporating Xpert for initial detection and LPA or DST for resistance confirmation, has the potential to improve diagnostic accuracy and guide more targeted treatment decisions in high-burden regions. To strengthen evidence for such an approach, multi-center studies, genomic sequencing of discordant isolates, and comprehensive cost-effectiveness evaluations are needed to optimize TB diagnostic algorithms and inform control strategies in resource-limited settings.

## Appendices

Detailed phenotypic drug susceptibility testing procedures

Drug Preparation

Rifampicin vials were reconstituted with 4 mL of sterile water.

Aliquots of 150 µL were prepared and stored at -20°C until use.

Inoculum Preparation

Fresh cultures (<14 days old) of Mycobacterium tuberculosis were emulsified in 4 mL MGIT broth with glass beads and vortexed.

The suspension was allowed to settle, and the supernatant was serially transferred through two sterile tubes.

Final turbidity was adjusted to 0.5 McFarland standard, followed by a 1:5 dilution in sterile saline for drug tube inoculation.

MGIT Tube Inoculation

For each isolate, two 7 mL MGIT tubes were prepared: Growth control (GC): received 0.8 mL MGIT supplement and 0.5 mL of 1:100 suspension. Drug-containing tube: received 0.8 mL supernatant, 0.5 mL of 1:5 inoculum, and 100 µL of rifampicin solution.

Tubes were incubated at 37°C in the MGIT 960 system and monitored daily for up to 13 days.

Interpretation

Growth results were interpreted using manufacturer-recommended growth until cutoffs.

Positive and negative controls, including the *Mycobacterium tuberculosis* H37Rv strain, were run with every batch.
